# PROJECT ECHO-GERIATRICS EXPANDS CAPACITY FOR STUDENT INTERPROFESSIONAL EDUCATION

**DOI:** 10.1093/geroni/igae098.2166

**Published:** 2024-12-31

**Authors:** Katherine Bennett, Tracy Brazg, Ashley McPeak, Mayumi Willgerodt, Barbara Cochrane, Leigh Ann Mike, Elizabeth Phelan

**Affiliations:** University of Washington, Seattle, Washington, United States; University of Washington, Seattle, Washington, United States; University of Washington, Seattle, Washington, United States; University of Washington, Seattle, Washington, United States; University of Washington, Seattle, Washington, United States; University of Washington, Seattle, Washington, United States; University of Washington, Seattle, Washington, United States

## Abstract

Interprofessional collaborative practice training is important for all health professions trainees, particularly for those who will care for older adults. However, clinical interprofessional training opportunities are limited. The Extension for Community Healthcare Outcomes (ECHO) model trains health professionals in specialty care via case-based sessions with a virtual learning community. We sought to determine whether integration of health professions students into an existing Project ECHO-Geriatrics would be a feasible and effective way to deliver training on age-friendly and interprofessional care. Our novel Age-Friendly Healthcare Interprofessional Training Program used Project ECHO as the platform for longitudinal, interprofessional health professions student education. Students were recruited from health professions schools across the University of Washington. After a brief introduction to age-friendly care and Project ECHO, participants attended at least 3 ECHO sessions and post-session debriefs. In its first two years, the program trained 44 students from 9 health professions programs (e.g. nursing, medicine, pharmacy, physical therapy, social work, dentistry). One hundred percent of participants reported the program was meaningful and worth their time. All competency items (5-point scale, 5 high, 1 low) improved, with the greatest improvements in understanding roles on an interprofessional team (3.1 pre, 4.0 post, p<.001) and confidence for providing age-friendly care (2.2 pre, 4.0 post, p<.001). Participants reported an increased likelihood of focusing on geriatrics in their careers. Open-ended feedback highlighted increased geriatrics knowledge as well as the importance of interprofessional teamwork. Project ECHO is a feasible and effective approach to rapidly expand age-friendly, interprofessional training for health professions students.

